# Effectiveness of a single-session early psychological intervention for children after road traffic accidents: a randomised controlled trial

**DOI:** 10.1186/1753-2000-4-7

**Published:** 2010-02-08

**Authors:** Daniel Zehnder, Martin Meuli, Markus A Landolt

**Affiliations:** 1 Department of Psychosomatics and Psychiatry, University Children's Hospital Zurich, Zurich, Switzerland; 2 Department of Surgery, University Children's Hospital Zurich, Zurich, Switzerland

## Abstract

**Background:**

Road traffic accidents (RTAs) are the leading health threat to children in Europe, resulting in 355 000 injuries annually. Because children can suffer significant and long-term mental health problems following RTAs, there is considerable interest in the development of early psychological interventions. To date, the research in this field is scarce, and currently no evidence-based recommendations can be made.

**Methods:**

To evaluate the effectiveness of a single-session early psychological intervention, 99 children age 7-16 were randomly assigned to an intervention or control group. The manualised intervention was provided to the child and at least one parent around 10 days after the child's involvement in an RTA. It included reconstruction of the accident using drawings and accident-related toys, and psychoeducation. All of the children were interviewed at 10 days, 2 months and 6 months after the accident. Parents filled in questionnaires. Standardised instruments were used to assess acute stress disorder (ASD), posttraumatic stress disorder (PTSD), depressive symptoms and behavioural problems.

**Results:**

The children of the two study groups showed no significant differences concerning posttraumatic symptoms and other outcome variables at 2 or at 6 months. Interestingly, analyses showed a significant intervention × age-group effect, indicating that for preadolescent children the intervention was effective in decreasing depressive symptoms and behavioural problems.

**Conclusions:**

This study is the first to show a beneficial effect of a single-session early psychological intervention after RTA in preadolescent children. Therefore, an age-specific approach in an early stage after RTAs may be a promising way for further research. Younger children can benefit from the intervention evaluated here. However, these results have to be interpreted with caution, because of small subgroup sizes. Future studies are needed to examine specific approaches for children and adolescents. Also, the intervention evaluated here needs to be studied in other groups of traumatised children.

**Trial Registration:**

Clinical Trial Registry: ClinicalTrials.gov: NCT00296842.

## Background

Road traffic accidents (RTAs) represent the leading health threat to children in industrialised countries [1]. Each year in Europe, approximately 9000 children and adolescents under the age of 19 die in an RTA, and 355 000 are injured [2]. The number of collisions without physical injury is probably considerably higher. There is sound evidence today that children can suffer significant and long-lasting psychological distress following RTAs. Previous studies report that about 10% to 30% of traffic-injured children develop acute stress disorder (ASD) in the first four weeks after an RTA [3-5]. Posttraumatic stress disorder (PTSD) or clinically relevant posttraumatic stress symptoms (PTSS) are found in up to 35% of injured children several months to years after an RTA [1,3,4,6-9]. In addition, studies report clinically relevant depressive symptoms and accident-related anxieties in about 15% to 25% of affected children several months after an RTA [3,6]. In some studies, girls have been shown to have a higher risk for PTSD than boys [9,10]. In most studies, age was not associated with PTSD [4,5,8,10]. A recent study [11] identified early PTSS as a significant predictor of low quality of life one year after an RTA in children; the researchers concluded that the return of injured children to pre-injury quality of life may therefore also depend on awareness and timely interventions regarding PTSS.

As a consequence of these significant and long-term mental health problems, there is considerable interest in early psychological interventions for RTA victims to prevent future symptoms. For adults, psychological debriefing is the most common intervention in the initial days after trauma exposure. This highly standardised approach, also known as Critical Incident Stress Debriefing (CISD) [12], aims to prevent or ameliorate adverse psychological long-term reactions. But a recent Cochrane review [13] on the efficacy of CISD in adults found no evidence that single-session individual debriefing prevented the onset of PTSD or reduced psychological distress. However, in children the research on the efficacy of single-session early interventions is not yet conclusive because there is just one previous RCT on this issue. The question as to whether more targeted and multiple session interventions in high-risk persons make more sense not only with adults [14] but also with children can be answered only by methodologically strong studies with children and adolescents. For use of CISD with acutely traumatised children several researchers modified the debriefing procedure [15,16]. Comparable to the procedure in adults, most research groups recommended reconstruction of the traumatic event. Some used drawings and trauma-related toys in order to explore the traumatic event not only verbally. Further, previously described interventions with children also dealt with trauma-related appraisals and the emotional impact of the event. Moreover, psychoeducation on posttraumatic stress was often provided. It is interesting to note that previous studies on early interventions with traumatised children did not systematically involve parents, although several studies showed that parental factors are important predictors of psychological adjustment in the child [1,6,8,11,17].

Studies on the effectiveness of early interventions with children lack methodological soundness and included case reports [18,19] and uncontrolled trials [20-23]. To date, there is one controlled trial [17] and one randomised controlled trial [24] in which a psychological debriefing format was conducted with children after accidents. Kenardy et al. [17] evaluated an early, information-provision intervention with children (age 7-15 years) and their parents following paediatric accidental injury. Booklets given to the participants within 72 hours of the accident provided information on common responses to trauma and the common time course of symptoms and suggestions for minimising any stress symptoms. This intervention was delivered to one of two hospitals (N = 33); the second hospital was the control (N = 70). The authors showed that their intervention reduced child anxiety symptoms at 1-month follow-up and parental posttraumatic intrusion symptoms and overall posttraumatic symptoms at the 6-month follow-up. This psychoeducative intervention therefore appears to be beneficial to injured children and their parents. However, the researchers noted that randomised controlled trails with larger sample sizes are needed to confirm the efficacy of an intervention of this kind. The RCT by Stallard et al. [24] evaluated an early psychological intervention with children (N = 158) age 7-18 years four weeks after an RTA. The children in both the control group and the intervention group demonstrated considerable improvements in psychological symptoms such as PTSS, depression, anxiety and behavioural problems at follow-up 8 months later. However, the single-session early intervention did not result in any additional significant gains. Several reasons may have led to these findings. First, the duration of four weeks between the RTA and the intervention is probably too long, because PTSS may have already developed in some children. Second, in some children a late intervention may interfere negatively with the natural course of coping with the traumatic event. Third, the age range of the sample was very large, and developmental differences between younger children and adolescents were not considered. It is conceivable that a purely verbal debriefing could be too difficult for younger children. Fourth, parents were not involved in the intervention, although parental support has been shown to be important for the recovery of the child after a trauma [6]. Fifth, follow-up was limited to one assessment 8 months after the accident. Stallard et al. [24] declared that therefore variations in the speed of recovery between the groups may not have been detected.

In sum, previous research on early psychological interventions with children after RTAs and other forms of traumatic events is fragmentary, and most studies are limited by methodological shortcomings. Therefore, no evidence-based recommendations can be made regarding early psychological intervention with traumatised children.

The present study aimed at assessing the effects of a single-session early psychological intervention in school-age children after RTAs by means of a randomised controlled trial. Our basic idea was that an early intervention might have the potential to prevent future psychological symptoms. Specifically, we tried to overcome shortcomings of previous studies by applying a more age-appropriate intervention (not only verbally, but also with drawings and accident-related toys), by providing the intervention between 7 to 10 days after the RTA, by including the children's parents and by assessing outcome at two follow-ups within 6 months. We assumed this approach to be effective. In addition, we tried to find out if specific factors, such as age and sex of the child and the severity of baseline acute stress symptoms, had an influence on the effect of the intervention. Based on the literature on the effectiveness of trauma-focused cognitive-behavioural therapy (tf-CBT) in children [25] we hypothesised that none of these moderating factors would yield any significant main effects.

## Methods

### Participants

**Figure 1:**
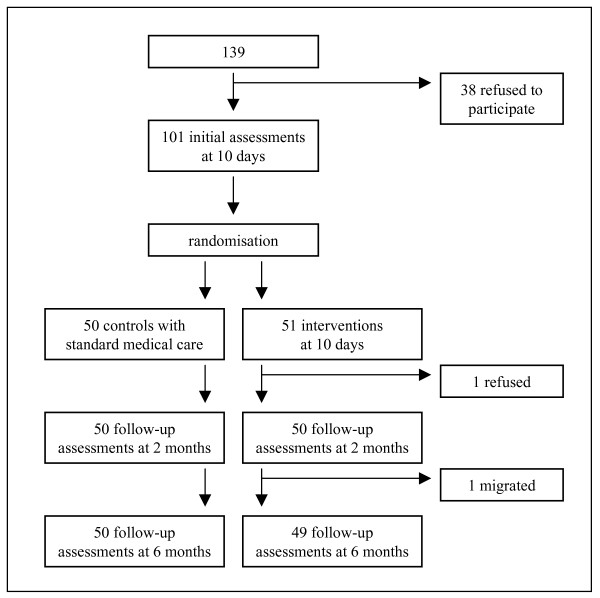
**CONSORT diagram of study cohort**.

Participants were recruited continuously from September 2004 until September 2007 at University Children's Hospital in Zurich, Switzerland. They had to meet all of the following criteria: (1) medical treatment (inpatient or outpatient) after an RTA (collision), (2) age between 7 and 16 years, (3) fluency in German, (4) no severe head injury (Glasgow Coma Scale >11), and (5) no previous evidence of intellectual impairment (according to medical records). Families with a child who met the criteria for inclusion were contacted within the first week after their child's accident; 139 children met the inclusion criteria and were asked to participate. Thirty-eight (16 boys, 22 girls) declined participation, mainly because the families had no interest in the study or because it seemed too time-consuming (Figure 1). Due to incomplete data at follow-up assessments, the final study sample comprised 99 children (response rate 71.2%). Comparison of participants and non-participants revealed no significant differences in mean age at accident (t = 0.19, p = .85), sex (χ^2 ^= 2.95, p = .09), type of accident (χ^2 ^= 1.45; p = .23) and mean injury severity (t = 1.07, p = .29).

### Procedure

The study was approved by the local institutional review board. Written informed consent was obtained from parents in agreement with the children. Assessments were carried out at around 10 days (T0), at 2 months (T1) and at 6 months (T2) after the child's involvement in an RTA. The children were assessed by means of a standardised, 30-45 minute interview conducted by trained psychologists. Most of the interviews were conducted in the participants' home; some were conducted at the hospital. Mothers were assessed at the same time using questionnaires. Medical variables were retrieved from the patients' records and the responsible physicians. In return for participation, families received 50 Swiss francs after completing all three assessments.

A priori power calculations were generated using GPower3 [26]. For an effect size of 0.60 and a power of 0.85, we aimed at a sample size of 102. The randomisation list, stratified for sex, was generated by the program RANCODE 3.6 (IDV, Gauting, Germany) at the beginning of the project. Blocks of 2 and 4 that alternated at random created similar sizes for both study groups. Immediately after the baseline assessment the interviewer opened an envelope that contained the predetermined randomisation for the particular child. If the child was assigned to the intervention group, the manualised intervention was conducted. Follow-up assessments at 2 and 6 months were conducted by a different interviewer, who was blind to the child's status in the project.

### Measures

#### Child ASD and PTSD

Accident-related acute and posttraumatic stress reactions were assessed using a standardised clinical interview, the IBS-KJ [27]. The two versions of this interview contain the criteria for ASD and PTSD according to the Diagnostic and Statistical Manual of Mental Disorders (DSM-IV-TR) [28]. The interview for PTSD (IBS-P-KJ) is a German version of the Clinician-Administered PTSD Scale for Children and Adolescents (CAPS-CA) [29]. This widely used diagnostic interview includes all symptoms of PTSD, scored on a 5-point frequency rating (Likert) scale (from 0 = none of the time to 4 = most of the time) and additionally on a 5-point intensity rating scale (from 0 = not a problem, none to 4 = a whole lot, very severe problem). The interview for ASD (IBS-A-KJ) was constructed similarly to assess DSM-IV-TR acute stress disorder symptoms. In the present study a total score was obtained for both instruments by summing across all items. In addition, ASD and PTSD were diagnosed according to the DSM-IV-TR. A symptom was considered present if the frequency was scored at least "1" and the intensity rating at least "2". Subsyndromal ASD/PTSD was diagnosed according to Bryant et al. [30,31] if criteria for one of the symptom clusters were not fulfilled. Previous studies supported the reliability and validity of this instrument [27,29]. In this study, internal consistencies of the IBS-KJ total score were found to be excellent, with Crohnbach's α of 0.94 at T0, 0.93 at T1 and 0.93 at T2.

#### Child depression

The presence of depressive symptoms was assessed using the German version (DIKJ) [32] of the Children's Depression Inventory [33]. For each item the child has three possible responses rating severity, from 0 = no symptoms, 1 = mild symptoms, to 2 = definite symptoms. A total score was obtained by summing across all 26 items. A cut-off of 18 points has been shown to identify children with clinically relevant depression [32]. Good psychometric properties of this instrument were reported [32]. For the current study Cronbach's α was 0.87 at T0, 0.83 at T1, and 0.85 at T2.

#### Child behavioural problems

Children's behavioural problems were assessed by the German version of the Child Behavior Checklist (CBCL) [34,35]. The CBCL is designed to record children's competencies and behavioural problems as reported by their parents. In this study, the questionnaire was completed by the children's mothers. The social competencies section was not included. The 120 items of the behavioural problems section are scored on a 3-point Likert scale ranging from 0 = not true to 2 = often true of the child. The CBCL contains eight problem syndrome scales as well as global scales for internalising, externalising, and total problems. All psychometric properties of this instrument were found to be acceptable [34]. In this study only the scale for total problems was used and transformed to T-scores that are based on a representative population of 2900 children and adolescents in Germany [35]. T-scores of 60 and more represent cases with clinically significant behavioural maladjustment. The CBCL showed excellent internal consistency in this sample (α = 0.93 at T0, α = 0.94 at T1 and α = 0.92 at T2).

#### Socio-economic status

Socio-economic status (SES) as assessed by mothers was calculated by means of a 6-point score of both paternal occupation and maternal education. The lowest SES score was 2 points, the highest 12 points. Three social classes were defined as follows: scores 2-5, lower class; scores 6-8, middle class; and scores 9-12, upper class. This measure was used in previous studies and was shown to be a reliable and valid indicator of SES in Switzerland [36].

#### Life events

We assessed the occurrence of 12 major life events (such as change of residence, unemployment in the family or parental separation) during the 12 months prior to the accident and the 6 months following the accident based on mothers' reports. A life event score was computed by summing up the number of life events for each family.

#### Severity of injuries

Severity of injuries was classified by a physician using the Modified Injury Severity Scale (MISS), a highly reliable and widely accepted scale [37]. The MISS values rate the severity of injuries in different bodily systems and range from 1 to 75, with scores >25 indicating severe injury.

### Intervention

At least one parent (71.4% mothers, 10.2% fathers, 18.4% both) was present at the intervention that lasted about 30 minutes. The intervention was short and therefore economic in order to have the chance of implementation within the routinely medical procedures of a children's hospital. The psychologist used a series of standard prompts systematically to guide the child through a structured, four-step process: (1) Detailed reconstruction of the accident and creation of a trauma narrative: Drawings and accident-related toys (e.g. figures, model cars, bicycles, etc.) were used as aids to talk about the course of the event in a concrete and age-appropriate way. (2) Identification of accident-related appraisals: The children were asked to report their thoughts about the traumatic event; if dysfunctional appraisals were mentioned, the psychologist assisted the child in modifying them. (3) Psychoeducation: Information on common stress reactions was given to normalise the child's early reactions. After that, the psychologist discussed with the child and the parents helpful strategies for dealing with acute stress reactions (such as talking about the accident at home, seeking social support, maintaining a daily routine, monitoring the symptoms). Parents were given special advice how to support their child in general. (4) Leaflet: As a last step, the child and the parents were given written information on posttraumatic stress and a contact address. For all of the participants, the intervention contained the same four steps, but the psychologist tried to adapt his language to the age of the child. Because all of the interventions were provided by the same psychologist, the procedure was identical for all of the 49 children and adolescents of the intervention group.

### Control condition

The children of the control group received standard medical care, including clinical diagnostics and comprehensive medical treatment. Different professionals (paediatricians, surgeons, physiotherapists, occupational therapist, etc.) were available if needed. Psychological support was also available but not routinely provided. In our sample, none of the participants received psychological support or treatment during the duration of the study.

### Statistical analyses

The data were analysed using the statistical package SPSS for Windows, release 16 (SPSS Inc., Chicago, IL). Analyses were performed with two-sided tests. χ^2 ^analyses were used to compare nominal variables. Normally distributed continuous data were analysed using independent t-tests (between-groups). To study the influence of the intervention over time, two factorial analyses of variance (ANOVAs) with repeated measures design were calculated. A series of additional analyses of covariance (ANCOVAs) were conducted entering age, sex and severity of baseline acute stress symptoms as main effects and the interactions with intervention condition to ascertain whether any of these characteristics might moderate differential responses to treatment. In all cases a p < .05 was considered significant. If significant mean differences were detected, effect sizes (d) were calculated following Cohen [38]. Kolmogorov-Smirnov Goodness of Fit Tests of the outcome variables showed normality for the IBS-KJ, the DIKJ and the CBCL.

## Results

### Sample characteristics and baseline assessment

Table 1 presents sample characteristics. There were no significant differences between the study groups on any demographic, accident or injury measure. Similarly, there were no significant between-group differences on any baseline score (T0) assessed at an average of 10.1 (SD = 3.0) days after the RTA (IBS-A-KJ: t = 0.64, p = .53; DIKJ: t = 0.42, p = .68; CBCL: t = 1.42, p = .16).

**Table 1:** Characteristics of the sample (N = 99)

	Intervention (N = 49)	Control group (N = 50)	t*	χ^2^†	p
**Mean (SD) age at accident, years**	11.8 (2.6)	11.3 (2.8)	0.77		.44

**Sex**					
Boys (%)	29 (59.2)	29 (58.0)			
Girls (%)	20 (40.8)	21 (42.0)		0.01	.91

**Socio-economic status**					
Lower (%)	6 (12.2)	4 (8.0)			
Middle (%)	16 (32.7)	19 (38.0)			
Upper (%)	24 (49.0)	22 (44.0)			
Unknown (%)	3 (6.1)	5 (10.0)		0.09	.77

**Mean (SD) number of preceding life events**	1.3 (1.6)	1.1 (1.4)	0.64		.53

**Mean (SD) number of life events that followed**	1.1 (1.5)	1.1 (1.8)	-0.15		.88

**Type of accident**					
Pedestrian (%)	16 (32.7)	18 (36.0)			
Car passenger (%)	8 (16.3)	7 (14.0)			
Bicycle/motorcycle (%)	17 (34.7)	17 (34.0)			
Other (%)	8 (16.3)	8 (16.0)		0.07	.79

**Mean (SD) score on the Modified Injury Severity Scale**	6.1 (4.6)	5.8 (5.3)	0.26		.80

**Medical treatment**					
Inpatient (%)	31 (63.3)	30 (60.0)			
Outpatient (%)	18 (36.7)	20 (40.0)		0.11	.74

*Independent two-sample t-test. †χ^2 ^analysis

The initial assessment identified 20 of the 99 children (20.2%) as meeting the diagnostic criteria for ASD (4.0%) or subsyndromal ASD (16.2%), 11 in the intervention group and 9 in the control group. This difference was not statistically significant (χ^2 ^= -0.47; p = .64). 13.1% of the children (5 in the intervention, 8 in the control group) had scores in the clinical range of depression, and 19.1% (11 in the intervention, 8 in the control group) showed clinically significant behavioural maladjustment, with no significant differences between the two study groups (DIKJ: χ^2 ^= 0.33; p = .56; CBCL: χ^2 ^= 1.69 p = .19).

### Follow-up assessments

The children were re-assessed at T1 at an average of 73.5 (SD = 14.7) days and at T2 at an average of 197.9 (SD = 20.6) days after the accident. No significant between-group differences were found at any time point for PTSS (T1: t = 0.81, p = .42; T2: t = 0.58, p = .57), depressive symptoms (T1: t = -0.34, p = .74; T2: t = -0.36, p = .72) or behavioural problems (T1: t = -0.01, p = .99; T2: t = -0.40, p = .69). ANOVA results for intervention and time variables (Table 2) showed significant improvements from T0 to T1 and T2 in both groups on PTSS, depressive symptoms and behavioural problems. Neither the intervention nor the interaction of time and intervention had significant influences on any outcome measure.

**Table 2:** Means, standard deviations and analysis of variance results for repeated measures

	Intervention (N = 49)	Control group (N = 50)	ANOVA F		
	
	M (SD)	M (SD)	Time (T)	Intervention (I)	T × I
**Traumatic stress symptoms: IBS-KJ**			32.90***	0.57	0.10
T0, total score	29.3 (23.7)	26.3 (23.0)			
T1, total score	21.6 (21.9)	18.5 (15.6)			
T2, total score	15.9 (19.3)	14.1 (11.2)			

**Depressive symptoms: DIKJ**			15.38***	0.76	0.01
T0, total score	10.1 (6.0)	9.6 (6.5)			
T1, total score	8.2 (5.8)	8.6 (5.8)			
T2, total score	7.2 (5.9)	7.7 (5.6)			

**Behavioural problems: CBCL**			9.41***	2.21	0.01
T0, T-score	53.4 (9.3)	50.6 (9.1)			
T1, T-score	50.0 (10.5)	50.0 (11.4)			
T2, T-score	47.4 (9.5)	48.2 (9.0)			

***p < = .001

Seven children (7.1%) at T1 and 4 children (4.0%) at T2 met the full diagnostic criteria for PTSD. In addition 7 children (7.1%) at T1 and 9 children (9.1%) at T2 fulfilled the criteria for subsyndromal PTSD. Again, there were no significant differences of these rates between the intervention group and the control group (T1: χ^2 ^= -0.45, p = .65; T2: χ^2 ^= -0.81, p = .42). Ten children (10.1%) at T1 and 4 children (4.0%) at T2 had scores in the clinical range of depression, without any significant differences between the study groups (T1: χ^2 ^= 0.17, p = .68; T2: χ^2 ^= 0.18, p = .67). Nineteen children (19.2%) at T1 and 10 children (10.1%) at T2 were clinically noticeable concerning behavioural problems. Again, no significant differences between the intervention group and the control group were found (T1: χ^2 ^= 0.00, p = .99; T2: χ^2 ^= 0.13, p = .72).

### Subgroup analyses

Three subgroup analyses were performed to examine if specific groups of children could profit from the intervention. Subgroups were constituted according to age, sex and severity of baseline acute stress symptoms.

Splitting the sample by age (median = 11.6 years) created a subgroup of 49 (27 intervention, 22 control) adolescents age 12-16 and 50 (22 intervention, 28 control) children age 7-11. In the older group no significant differences on any outcome measure could be found between the intervention and the control groups at T0, T1 or T2 (Table 3). In the younger half of the sample two of the three between-group differences at T2 were significant. The 7- to 11-year old children in the intervention group showed significant improvements from T0 to T2 on depression (effect size d = 0.99) and behavioural problems (d = 0.76). No such improvements were found in the control group (DIKJ: d = 0.15; CBCL: d = -0.02). However, the mean PTSS scores did not differ significantly between the intervention group and the control group at any time point. ANCOVAS of T2 scores as a function of intervention condition and age-group with T0 scores as covariates (Table 4) confirmed the influence of the interaction variable to depressive symptoms and behavioural problems at T2. This interaction of the intervention condition and age group was therefore significant for depression (DIKJ) and behaviour (CBCL) but not for the PTSS scores.

**Table 3:** Between group comparisons of mean (SD) scores at T0, T1 and T2 in subgroups according to age

	Intervention	Control group	t*	p
**Preadolescent children (7-11 years)**				
IBS-KJ, T0 (total scores)	27.7 (25.2)	24.9 (21.4)	0.43	.69
IBS-KJ, T1 (total scores)	22.2 (22.9)	17.4 (12.2)	0.96	.34
IBS-KJ, T2 (total scores)	14.9 (17.4)	15.0 (10.5)	-0.03	.98
DIKJ, T0 (total scores)	8.8 (5.1)	8.4 (5.7)	0.26	.79
DIKJ, T1 (total scores)	5.6 (3.4)	7.8 (4.5)	-1.83	.07
DIKJ, T2 (total scores)	4.5 (3.4)	7.6 (5.1)	-2.41	**.02**
CBCL, T0 (T-scores)	53.0 (10.3)	51.1 (8.8)	0.65	.52
CBCL, T1 (T-scores)	49.4 (12.6)	54.7 (10.2)	-1.41	.17
CBCL, T2 (T-scores)	45.1 (10.6)	51.3 (7.1)	-2.04	**.05**

**Adolescents (12-16 years)**				
IBS-KJ, T0 (total scores)	30.6 (22.8)	28.2 (25.2)	0.36	.72
IBS-KJ, T1 (total scores)	21.1 (21.5)	20.0 (19.4)	0.20	.85
IBS-KJ, T2 (total scores)	16.8 (21.1)	12.9 (12.2)	0.76	.45
DIKJ, T0 (total scores)	11.2 (6.6)	11.2 (7.2)	0.03	.98
DIKJ, T1 (total scores)	10.2 (6.6)	9.6 (7.1)	0.32	.75
DIKJ, T2 (total scores)	9.5 (6.6)	7.8 (6.2)	0.92	.36
CBCL, T0 (T-scores)	53.7 (8.5)	49.9 (9.6)	1.36	.18
CBCL, T1 (T-scores)	50.0 (9.6)	44.3 (10.3)	1.78	.08
CBCL, T2 (T-scores)	49.2 (8.4)	45.0 (9.8)	1.42	.16

*Independent two-sample t-test

**Table 4:** Analysis of covariance of T2 scores as a function of intervention condition and age group, with T0 scores as covariate

Source	df	MS	F	p
**Posttraumatic stress symptoms: IBS-KJ, T2**				
IBS-KJ, T0 (covariate)	1	7256.6	40.83	**<.001**
Intervention	1	19.2	0.11	.74
Age group	1	39.9	0.22	.64
Intervention × age group	1	104.7	0.59	.45
Error	94	177.7		

**Depressive symptoms: DIKJ, T2**				
DIKJ, T0 (covariate)	1	1153.3	61.82	**<.001**
Intervention	1	16.2	0.87	.35
Age group	1	28.5	1.53	.22
Intervention × age group	1	151.2	8.11	**.01**
Error	94	18.7		

**Behavioural problems: CBCL, T2**				
CBCL, T0 (covariate)	1	2637.6	60.3	**<.001**
Intervention	1	148.3	3.39	.07
Age group	1	2.8	0.06	.80
Intervention × age group	1	229.8	5.25	**.03**
Error	67	43.7		

Table 5 shows that there were no significant differences on any outcome measure between the two study groups at T0, T1 or T2 in the subgroup of the 41 girls (20 intervention, 21 control) and in the subgroup of the 58 boys (29 intervention, 29 control).

**Table 5:** Between group comparisons of mean (SD) scores at T0, T1 and T2 in subgroups according to sex

	Intervention	Control group	t*	p
**Girls**				
IBS-KJ, T0 (total scores)	40.2 (27.2)	35.8 (29.2)	0.50	.62
IBS-KJ, T1 (total scores)	29.9 (23.8)	24.6 (20.4)	0.76	.45
IBS-KJ, T2 (total scores)	21.3 (20.6)	15.9 (13.8)	0.99	.33
DIKJ, T0 (total scores)	11.7 (6.4)	11.9 (7.6)	-0.06	.95
DIKJ, T1 (total scores)	9.3 (6.6)	10.4 (6.5)	-0.53	.60
DIKJ, T2 (total scores)	8.0 (5.7)	7.7 (5.4)	0.14	.89
CBCL, T0 (T-scores)	55.2 (9.5)	50.3 (9.8)	1.46	.15
CBCL, T1 (T-scores)	53.5 (7.9)	50.9 (12.9)	0.65	.52
CBCL, T2 (T-scores)	49.0 (6.7)	48.5 (9.4)	0.16	.88

**Boys**				
IBS-KJ, T0 (total scores)	21.9 (17.8)	19.5 (14.2)	0.56	.58
IBS-KJ, T1 (total scores)	15.9 (18.8)	14.1 (9.1)	0.47	.64
IBS-KJ, T2 (total scores)	12.2 (17.8)	12.8 (8.9)	-0.15	.88
DIKJ, T0 (total scores)	9.0 (5.6)	8.0 (5.1)	0.76	.45
DIKJ, T1 (total scores)	7.4 (5.2)	7.2 (4.9)	0.11	.92
DIKJ, T2 (total scores)	6.8 (6.2)	7.6 (5.8)	-0.55	.59
CBCL, T0 (T-scores)	52.3 (9.2)	50.8 (8.8)	0.62	.54
CBCL, T1 (T-scores)	47.9 (11.4)	49.4 (10.5)	0.48	.63
CBCL, T2 (T-scores)	46.3 (11.0)	48.0 (8.8)	-0.55	.58

*Independent two-sample t-test

In a subgroup of 19 children with diagnosed ASD or subsyndromal ASD at T0 (9 intervention, 12 control) no significant differences could be found on any outcome measure between the intervention group and the control group at any time point (Table 6). Likewise, no significant differences between the study groups were found in the subgroup of the 78 remaining children (40 intervention, 38 control) without any diagnosis of ASD.

**Table 6:** Between group comparisons of mean (SD) scores at T0, T1 and T2 in subgroups according to severity of baseline acute stress symptoms

	Intervention	Control group	t*	p
**ASD/subsyndromal ASD at T0**				
IBS-KJ, T0 (total scores)	59.6 (14.5)	47.8 (23.0)	1.34	.20
IBS-KJ, T1 (total scores)	42.3 (23.5)	27.8 (16.2)	1.69	.11
IBS-KJ, T2 (total scores)	30.6 (22.5)	16.6 (10.8)	1.89	.07
DIKJ, T0 (total scores)	14.7 (4.1)	13.6 (6.7)	0.46	.65
DIKJ, T1 (total scores)	11.8 (5.6)	11.5 (7.1)	0.10	.92
DIKJ, T2 (total scores)	8.4 (4.3)	9.8 (6.4)	-0.56	.58
CBCL, T0 (T-scores)	49.7 (14.7)	53.0 (7.5)	-0.51	.63
CBCL, T1 (T-scores)	46.6 (13.0)	52.9 (14.6)	-0.80	.44
CBCL, T2 (T-scores)	46.3 (8.9)	48.0 (7.0)	-0.38	.71

**No ASD at T0**				
IBS-KJ, T0 (total scores)	22.5 (19.7)	19.6 (18.5)	0.69	.50
IBS-KJ, T1 (total scores)	17.0 (18.8)	15.6 (14.5)	0.35	.73
IBS-KJ, T2 (total scores)	12.7 (17.2)	13.3 (11.3)	-0.20	.84
DIKJ, T0 (total scores)	9.1 (6.0)	8.3 (6.0)	0.57	.57
DIKJ, T1 (total scores)	7.4 (5.6)	7.6 (5.1)	-0.23	.82
DIKJ, T2 (total scores)	7.0 (6.2)	7.0 (5.2)	0.00	.99
CBCL, T0 (T-scores)	53.9 (8.3)	50.0 (9.4)	1.90	.06
CBCL, T1 (T-scores)	50.5 (10.2)	49.1 (10.4)	0.53	.60
CBCL, T2 (T-scores)	47.6 (9.7)	48.3 (9.5)	-0.29	.77

*Independent two-sample t-test

## Discussion

The present study is the second randomised controlled trial of a single-session early psychological intervention for child survivors of RTAs and the first to show a beneficial effect in preadolescent children.

In the overall sample, our results demonstrated that children in both the intervention and the control groups made the same significant improvements with regard to PTSS, depressive symptoms and behavioural problems between 10 days and 6 months after the RTA. Therefore, the intervention had no beneficial effect on the course of the symptoms in the overall sample. This contradicts our hypothesis but is in line with the study by Stallard et al. [24] and consistent with the conclusion of the recent Cochrane review concerning adults [13]. On the other hand, it is important to point out that we found no evidence of harmful effects of our intervention, a finding that several studies reported for traumatised adults [13]. Several reasons may have contributed to the inefficiency of our single-session intervention. First, the early contact with the child and the family and the highly structured assessment with the participants of both study groups may in itself have been therapeutic by acknowledging, validating and normalising the child's symptoms [24]. Second, our intervention may have been too short. It is perhaps hardly possible to generate sustainable effects in only one session. Notably, in adults, early interventions proved to be effective only if multiple sessions are conducted [14]. Third, it might be possible that our control condition reflects a high standard of medical care with a generally good aftercare by paediatricians. Fourth, it must be considered that an intervention in an early stage after a traumatic event could interfere with natural coping mechanisms or disrupt an adaptive defence mechanism [24].

It is an interesting finding that the intervention was effective in children age 7-11 years by significantly reducing depressive symptoms and behavioural problems. This is in contrast to the results of the previous RCT study by Stallard et al. [24] and may be explained by the following differences in methodology: First, in our study the intervention took place at a much earlier stage (10 days after the RTA). The clinical experiences of the psychologist conducting the intervention showed that all of the children had overcome the initial shock at this point and were ready to deal with the RTA in detail. Second, our intervention included at least one parent. This might be particularly important for helping younger children to feel safe. Moreover, during the intervention the parents experienced open communication by the psychologist regarding the accident. It is conceivable that this could have increased the impact of the intervention due to a positive influence on increased openness in future parent-child communication. Third, the reconstruction of the accident and the creation of a trauma narrative by means of drawings and accident-related toys were well-suited to the cognitive stage of development in younger children. They may have more difficulty with purely verbal interventions as provided by Stallard et al. [24] and may benefit from a more age-appropriate intervention. As previous studies showed, it was difficult for young children to talk about stress symptoms, but drawings facilitated the children's verbal reports of emotionally laden events [39,40].

Even if in both study groups the total scores of the IBS-KJ improved within 6 months after RTAs, in this study the rates of children that met the DSM-IV-TR criteria for PTSD or subsyndromal PTSD remained at approximately 13%. This is in line with previous prospective studies [3,8] that found a high risk for chronic manifestations of PTSD following RTAs. However, the rates for ASD and PTSD are low in this study compared to the findings of international studies with child RTA victims [1,3-9]. We assume that methodical differences between the questionnaires used (clinical interview vs. self-report scale) might have caused these results. Also, studies in Swiss adult RTA victims have previously shown remarkable low rates of PTSD [41]. This fact could be an indicator of a well-functioning health system in general.

### Limitations

Several limitations of this study need to be addressed. First, subgroup analyses should be interpreted with caution, because of small subgroup sizes. Second, families with a lower socio-economic background were underrepresented in the sample, because families with no command of the German language were excluded. A third issue potentially limiting the generalisation of our findings is the participation rate of around 70%. Although this response rate was quite high, non-participation may be a consequence of ASD-related avoidance symptoms. On the other hand, it might be possible that some of the non-participants declined participation because they were well adjusted and the study was not relevant to them. Both cases would affect prevalence estimates of ASD and PTSD in this population. However, we do not think that this issue influenced the results regarding the effectiveness of our intervention. Besides, the small dropout-rate of 2% has to be pointed out. Fourth, the clinical significance of the intervention in the younger age group may be questioned, because the DIKJ and CBCL scores were not in a clinical range. The German norms of the DIKJ and CBCL questionnaires are perhaps not entirely suitable for the Swiss population and/or may not reflect today's situation because they were assessed in 2000 [32] and 1998 [35], respectively. Nevertheless, it is important to consider that the effect sizes of the improvements from T0 to T2 on depression and behavioural problems were quite high for the preadolescent children of the intervention group. Therefore, this progression of decreasing symptomatology is relevant for a particular child, even if the mean scores do not indicate clinical significance. Moreover, it may be hypothesised that the intervention effects may have been even larger in a sample with higher symptomatology.

### Clinical implications

Despite these limitations, the present study has several strengths, including its randomised controlled prospective design, the highly standardised assessment instruments, manualised intervention and very low dropout-rate. Moreover, statistical conditions were good, with no socio-demographic differences between study participants and non-participants and no differences in all baseline scores between intervention and control groups.

Our findings suggest using an age-specific and development-specific approach for dealing with traumatic symptoms in an early stage after an RTA. Young children can profit from a single-session early intervention around 10 days after an RTA. We suggest involving at least one parent during the intervention. The trauma narrative should be created with the aid of drawings and accident-related toys, which aid talking with the child in a concrete and adapted way. Furthermore, psychoeducational information on posttraumatic stress and possible ways to cope with PTSS should be discussed [22]. In adolescents a single-session early psychological intervention was not demonstrated to be effective. As results in adults showed [14], it may be useful for adolescents to be screened for ASD carefully in an early stage after an RTA. For adolescent trauma victims with low symptom scores, psychological interventions may not be necessary, and watchful waiting may be a better strategy. For adolescents with high symptom scores and their families, three to five sessions of tf-CBT may be appropriate to treat PTSS. In addition to the assessment of PTSS, depressive symptoms, anxieties and behavioural problems should be observed and treated carefully.

Further research is required to examine the differences between younger children and adolescents or adults. In general, early psychological interventions with victims of different traumatic events are greatly needed to prevent chronic suffering and to minimise subsequent economic costs.

## Conclusions

In this study, a single-session early psychological intervention was effective in preventing depressive symptoms and behavioural problems among preadolescent children after traffic accidents. Because adolescents did not benefit from the intervention, our findings suggest an age- and development-specific approach for dealing with traumatic symptoms in an early stage after a road traffic accident. Also, the intervention evaluated here needs to be studied in other groups of traumatised children.

## Competing interests

The authors declare that they have no competing interests.

## Authors' contributions

This work bases on DZ's doctoral dissertation at the University of Zurich, Zurich, Switzerland. DZ was involved in data collection, conducted the interventions, performed the data analysis and drafted the manuscript. MM participated in the design of the study and the acquisition and interpretation of data. MAL was DZ's doctoral advisor. MAL conceived the study, developed the research design, supervised all aspects of study and was involved in the writing of the paper. All authors read and approved the final version of the report.

## References

[B1] de Vries AP Kassam-Adams N Cnaan A Sherman-Slate E Gallagher PR Winston FK Looking beyond the physical injury: posttraumatic stress disorder in children and parents after pediatric traffic injury Pediatrics 1999 104 1293 1299 10.1542/peds.104.6.1293 10585980

[B2] European Child Safety Alliance Childhood road safety: facts 2007 Amsterdam: EuroSafe

[B3] Di Gallo A Barton J Parry-Jones W Road traffic accidents: early psychological consequences in children and adolescents Br J Psychiatry 1997 170 358 362 10.1192/bjp.170.4.358 9246255

[B4] Kassam-Adams N Winston FK Predicting child PTSD: the relationship between acute stress disorder and PTSD in injured children J Am Acad Child Adolesc Psychiatry 2004 43 403 411 10.1097/00004583-200404000-00006 15187800

[B5] Meiser-Stedman R Dalgleish T Smith E Yule W Bryant B Ehlers A Mayou RA Kassam-Adams N Winston F Dissociative symptoms and the acute stress disorder diagnosis in children and adolescents: a replication of the Harvey and Bryant (1999) study J Trauma Stress 2007 20 359 364 10.1002/jts.20211 17597127

[B6] Keppel-Benson JM Olledick TH Benson MJ Post-traumatic stress in children following motor vehicle accidents J Child Psychol Psychiatry 2002 43 203 212 10.1111/1469-7610.00013 11902599

[B7] Landolt MA Vollrath M Ribi K Gnehm HE Sennhauser FH Incidence and associations of parental and child posttraumatic stress symptoms in pediatric patients J Child Psychol Psychiatry 2003 44 1199 1207 10.1111/1469-7610.00201 14626460

[B8] Landolt MA Vollrath M Timm K Gnehm HE Sennhauser FH Predicting posttraumatic stress symptoms in children after road traffic accidents J Am Acad Child Adolesc Psychiatry 2005 44 1276 1283 10.1097/01.chi.0000181045.13960.67 16292120

[B9] Stallard P Velleman R Baldwin S Prospective study of post-traumatic stress disorder in children involved in road traffic accidents BMJ 1998 317 1619 1623 9848900 10.1136/bmj.317.7173.1619 PMC28739

[B10] Bryant B Mayou R Wiggs L Ehlers A Stores G Psychological consequences of road traffic accidents for children and and their mothers Psychol Med 2004 43 335 346 10.1017/S0033291703001053 14982139

[B11] Landolt MA Vollrath ME Gnehm HE Sennhauser FH Posttraumatic stress impacts on health-related quality of life in children after road traffic accidents: a prospective study Aust N Z J Psychiatry 2009 43 746 753 10.1080/00048670903001919 19629796

[B12] Mitchell JT When disaster strikes the critical incident stress debriefing process JEMS 1983 8 36 39 10258348

[B13] Rose S Bisson J Churchill R Wessely S Psychological debriefing for preventing post traumatic stress disorder (PTSD) Cochrane Database of Systematic Reviews 2008 1

[B14] Ehlers A Clark DM Early psychological interventions for adult survivors of trauma: a review Biol Psychiatry 2003 53 817 826 10.1016/S0006-3223(02)01812-7 12725974

[B15] Dyregrov A Grief in children: a handbook for adults 1991 London: Jessica Kingsley

[B16] Stallard P Salter E Psychological debriefing with children and young people following traumatic events Clin Child Psychol Psychiatry 2003 8 445 457 10.1177/13591045030084003

[B17] Kenardy J Thompson K Le Brocque R Olsson K Information-provision intervention for children and their parents following pediatric accidental injury Eur Child Adolesc Psychiatry 2008 17 316 325 10.1007/s00787-007-0673-5 18350366

[B18] Klingmann A A school-based emergency crisis intervention in a mass school disaster Prof Psychol Res Pract 1987 18 604 612 10.1037/0735-7028.18.6.604

[B19] Pynoos RS Eth S Witness to violence: the child interview J Am Acad Child Adolesc Psychiatry 1986 25 306 319 10.1016/S0002-7138(09)60252-1

[B20] Casswell G Learning from the aftermath: the response of mental health workers to a school bus crash Clin Child Psychol Psychiatry 1997 2 517 523 10.1177/1359104597024005

[B21] Poijula S Wahlberg KE Dyregrov A Adolescent suicide and suicide contagion in three secondary schools J Emerg Ment Health 2001 3 163 168 11642194

[B22] Vila G Porche LM Mouren-Simeoni MC An 18-month longitudinal study of posttraumatic disorders in children who were taken hostage in their school Psychosom Med 1999 61 746 754 10593625 10.1097/00006842-199911000-00005

[B23] Yule W Posttraumatic stress disorder in child survivors of shipping disaster: the sinking of the "Jupiter" Psychother Psychosom 1992 57 200 205 1410197 10.1159/000288599

[B24] Stallard P Velleman R Salter E Howse I Yule W Taylor G A randomised controlled trial to determine the effectiveness of an early psychological intervention with children involved in road traffic accidents J Child Psychol Psychiatry 2006 47 127 134 10.1111/j.1469-7610.2005.01459.x 16423143

[B25] Cohen JA Deblinger E Mannarino AP Steer RA A multisite, randomized controlled trial for children with sexual abused-related PTSD symptoms J Am Acad Child Adolesc Psychiatry 2004 43 393 402 10.1097/00004583-200404000-00005 15187799 PMC1201422

[B26] Erdfelder E Faul F Buchner A GPOWER: a general power analysis program Behav Res Meth Instrum Comput 1996 28 1 11

[B27] Steil R Füchsel G IBS-KJ: Interviews zu Belastungsstörungen bei Kindern und Jugendlichen 2005 Göttingen: Hogrefe

[B28] American Psychiatric Association The diagnostic and statistical manual of mental disorders. Text revision 1994 4 Washington, DC: American Psychiatric Association

[B29] Nader KO Kriegler JA Blake DD Pynoos RS Newman E Weather FW The Clinician-Administered PTSD Scale, Child and Adolescent Version (CAPS-CA) 2002 White River Junction: National Center for PTSD

[B30] Bryant RA Harvey AG Delayed-onset posttraumatic stress disorder: a prospective evaluation Aust N Z J Psychiatry 2002 36 205 209 10.1046/j.1440-1614.2002.01009.x 11982541

[B31] Bryant RA Salmon K Sinclair E Davidson P A prospective study of appraisals in childhood posttraumatic stress disorder Behav Res Ther 2007 45 2502 2507 10.1016/j.brat.2007.04.009 17560541

[B32] Stiensmeier-Pelster J Schürmann M Duda K Depressionsinventar für Kinder und Jugendliche; DIKJ 4 2000 Auflage. Göttingen: Hogrefe

[B33] Kovaks M The Children's Depression Inventory (CDI) Psychopharmacol Bull 1985 21 995 999 4089116

[B34] Achenbach TM Manual for the child behavior checklist 4-18 and 1991 profile 1991 Burlington, VT: University of Vermont

[B35] Doepfner M Plück J Bölte S Lenz K Melchers P Heim K Elternfragebogen über das Verhalten von Kindern und Jugendlichen. Deutsche Bearbeitung der Child Behavior Checklist (CBCL/4-18) 1998 Köln: KJFD

[B36] Landolt MA Vollrath M Ribi K Predictors of coping strategy selection in paediatric patients Acta Paediatr 2002 91 954 960 10.1080/080352502760272650 12412872

[B37] Mayer T Matlak ME Johnson DG Walker ML The Modified Injury Severity Scale in pediatric multiple trauma patients J Pediatr Surg 1980 15 719 726 10.1016/S0022-3468(80)80271-5 7463271

[B38] Cohen J Statistical power analysis for the behavioral sciences 1988 2 Hilsdale, NJ: Erlbaum

[B39] Gross J Hayne H Drawing facilitates children's verbal reports of emotionally laden events J Exp Psychol Appl 1998 4 163 174 10.1037/1076-898X.4.2.163

[B40] Meiser-Stedman R Smith P Glucksman E Yule W Dalgleish T The posttraumatic stress disorder diagnosis in preschool- and elementary school-age children exposed to motor vehicle accidents Am J Psychiatry 2008 165 1326 1337 10.1176/appi.ajp.2008.07081282 18676592

[B41] Schnyder U Wittmann L Friedrich-Perez J Hepp U Moergeli H Posttraumatic stress disorder following accidental injury: rule or exception in Switzerland? Psychother Psychosom 2008 77 111 118 10.1159/000112888 18230944

